# Benzamide Trimethoprim Derivatives as Human Dihydrofolate Reductase Inhibitors—Molecular Modeling and In Vitro Activity Study

**DOI:** 10.3390/biomedicines12051079

**Published:** 2024-05-13

**Authors:** Danuta Drozdowska, Agnieszka Wróbel-Tałałaj, Cezary Parzych, Artur Ratkiewicz

**Affiliations:** 1 Department of Organic Chemistry, Medical University of Białystok, Mickiewicza Street 2A, 15-222 Białystok, Poland; agnieszkawrobel9@gmail.com; 2 Department of Physical Chemistry, Faculty of Chemistry, University of Bialystok, Ciołkowskiego 1K Street, 15-245 Białystok, Poland; kab00m2510@gmail.com (C.P.); artrat@uwb.edu.pl (A.R.)

**Keywords:** DHFR inhibitors, anticancer activity, benzamides, drug design, trimethoprim derivatives

## Abstract

Human dihydrofolate reductase (*h*DHFR) is an essential cellular enzyme, and inhibiting its activity is a promising strategy for cancer therapy. We have chosen the trimethoprim molecule (**TMP**) as a model compound in our search for a new class of *h*DHFR inhibitors. We incorporated an amide bond, a structural element typical of netropsin, a ligand that binds selectively in the minor groove of DNA, into the molecules of **TMP** analogs. In this work, we present previously obtained and evaluated eleven benzamides (**JW1**–**JW8**; **MB1**, **MB3**, **MB4**). Recently, these compounds were specifically projected as potential inhibitors of the enzymes acetylcholinesterase (AChE) and β-secretase (BACE1). **JW8** was most active against AChE, with an inhibitory concentration of AChE IC_50_ = 0.056 µM, while the IC_50_ for donepezil was 0.046 µM. This compound was also the most active against the BACE1 enzyme. The IC_50_ value was 9.01 µM compared to that for quercetin, with IC_50_ = 4.89 µM. All the benzamides were active against *h*DHFR, with IC_50_ values ranging from 4.72 to 20.17 µM, and showed activity greater than **TMP** (55.26 µM). Quantitative results identified the derivatives **JW2** and **JW8** as the most promising. A molecular modeling study demonstrates that **JW2** interacts strongly with the key residue Gly-117, while **JW8** interacts strongly with Asn-64 and Arg-70. Furthermore, **JW2** and **JW8** demonstrate the ability to stabilize the *h*DHFR enzyme, despite forming fewer hydrogen bonds with the protein compared to reference ligands. It can be concluded that this class of compounds certainly holds great promise for good active leads in medicinal chemistry.

## 1. Introduction

Cancer remains one of the leading causes of death in the world, and as a result, there is a pressing need for the development of novel and effective treatments. Each new year, the FDA approves the use of new drugs for cancer treatments, but these are often challenged by multiple drug resistance and serious side effects [1]. The goal of cancer chemotherapy is to provide a cytotoxic effect to prevent tumor growth. Nowadays, drugs that cause damage to DNA or inhibit DNA synthesis by antimetabolite characteristics that may, such as inhibition of topoisomerase, inhibition of the key tyrosine kinases, and prevention of cell division through microtubule inhibition, are used in cancer treatment [2].

Dihydrofolate reductase (DHFR) is an indispensable enzyme required for the survival of most prokaryotic and eukaryotic cells, as it is involved in the biosynthesis of essential cellular components. DHFR has attracted a lot of attention as a molecular target for various diseases like bacterial infection, malaria, tuberculosis, dental caries, trypanosomiasis, leishmaniasis, fungal infection, influenza, Buruli ulcer, and respiratory illness [3]. Folate metabolism plays a key role in the biosynthesis of nucleic acid precursors and is still recognized as an important and attractive target for cancer chemotherapy [4]. This enzyme is becoming increasingly important in the design of new anticancer drugs, as confirmed by numerous studies, including modeling, synthesis, and in vitro biological research. Various DHFR inhibitors are being developed by many research teams to test their therapeutic efficacy. The main groups among the described compounds, DHFR inhibitors with anticancer properties, were methotrexate analogs, benzodiazepine derivatives, and 1,3,5-triazines. Reports on trimethoprim derivatives were few but confirmed that this molecule could be a model for further exploration of DHFR inhibitors [5].

**Figure 1:**
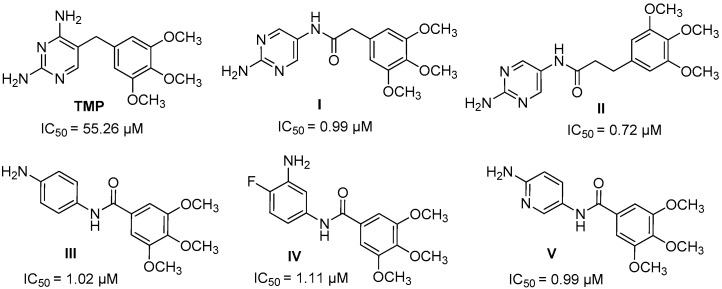
Structures of trimethoprim and its derivatives.

So, the trimethoprim molecule **TMP** (Figure 1) was chosen as a model structure in our search for new active DHFR inhibitors. In these studies, the pyrimidine ring in the **TMP** molecule was replaced by a benzene ring with different substitutes, and an amide bond was inserted in the place of the methylene bridge, allowing binding to a minor groove of DNA.

The first group of investigated compounds were those in which we presented the effect of replacing or lengthening the methylene bridge with an amide bond and lengthening the linker connecting the two aromatic rings by additional carbon atoms relative to the model structure of **TMP** [6]. These results confirmed our assumption of double activity of the synthesized compounds: DNA-binding effect and DHFR inhibitory activity, which was proven in vitro and by molecular docking. Compounds **I**, **II**, and **III** (Figure 1) were the most active and had similar activity to another DHFR inhibitor, methotrexate (IC_50_ = 0.08 µM), a potent and widely used anticancer agent usually included in therapeutic protocols for many different cancers. The results obtained from the molecular docking experiment show that the introduction of an amide bond into **TMP** analogs increases their affinity for human DHFR compared to unmodified **TMP**. Furthermore, molecular docking studies show that replacing the pyrimidine ring with a benzene ring slightly reduces the affinity for the enzyme, but derivatives of this type still interact with its key residues, that is, Glu-30 and Phe-34. The in vitro experimental findings revealed that all the newly designed and synthesized compounds exhibited higher activity against the DHFR enzyme and higher binding affinity than standard **TMP**. These results confirmed our assumption of the double activity of the synthesized compounds: DNA binding effect and DHFR inhibitory activity [6].

Based on the results of the first group of compounds, we designed eighteen new **TMP** derivatives to investigate the structure–activity relationship (SAR) and obtain more active DHFR inhibitors [7]. All synthesized **TMP** analogs had an amide bond. Our aim was to investigate the effect on the biological activity of the type of ring (benzene, pyrimidine, and pyridine), the position of the benzene ring, and the nature of the substituents. -I, -Cl, -F, -NH_2_, -OCH_3_, and some of them also contained carbon–carbon double bonds together with an amide bond linking the two aromatic rings. The ethidium bromide test showed that the tested **TMP** analogs bound to plasmid DNA more than the netropsin used as a standard. Trimethoprim does not bind to the plasmid and does not displace ethidium bromide from the complex with DNA.

Most of the derivatives obtained showed greater *h*DHFR inhibitory activity than trimethoprim, but none was more active than methotrexate **MTX**. Of this library, substances **IV,** bearing a benzene ring (4-F, 3-NH_2_), and **V,** with a pyridine ring, were the most active (Figure 1) against *h*DHFR; compounds with a double bond and cinnamic acid derivatives had similar activity. The introduction of a chlorine substituent at position 3 of the benzene ring did not increase the inhibitory activity of *h*DHFR, nor did a reduction in the number of methoxy groups at the ring. **TMP** derivatives with 3′,5′-dimethoxy-, 3′4′-dimethoxy-, or 3′-methoxybenzene in the structure were significantly less active than those with embedded 3′,4′,5′-trimethoxybenzene [7].

The search for active **TMP** derivatives is supported by molecular modeling methods. Results from the molecular docking study show that the most favorable position for molecules **IV** and **V** is the carboxylate group of Glu-30, which acts as an anchor point for the 2-amino group in inhibitor molecules **IV**. A wide range of different types of interactions were observed for this molecule, namely hydrogen bonding, interactions involving π orbitals, alkyl hydrophobic interactions, and halogen bonding. The binding mode of molecule **V** does not seem to directly involve interaction with Glu-30, but a strong contact takes place in its very close vicinity [7].

We found that molecules **IV** and **V** had high binding affinity to *h*DHFR (−8.0 kcal/mol). These compounds formed hydrogen bonds with Ser-59, Tyr-121, and Thr-146, with Glu-30 in the case of **IV**, and with Ala-9 in the case of compound **V**. Additionally, a π–π interaction was also observed between this group of ligands and the Phe-34 residue. Furthermore, an amide-π-type contact was evident between the aromatic ring of the ligand and the main chain of the Val-8 residue. In the case of derivative **IV**, there is also a halogen bond between the fluorine and Ile-7, but their effect on the binding energy is considered negligible. In addition, several different hydrophobic interactions were found with Ala-9, Ile-16, and Leu-22. The amide group plays an important role in binding the ligand but also elongates the chain between two aromatic rings, which makes interaction with positively charged Lys-55 possible [7].

Benzamides continue to be synthesized and extensively researched due to their wide range of applications, which include pharmaceuticals and medicinal chemistry [8,9] or polymeric materials [10]. Such compounds are useful building blocks in organic synthesis, and most methods focus on the formation of amide bonds, which are involved in almost all biological processes and are, therefore, ubiquitous [11] and are useful intermediates for the synthesis of various biologically active molecules. These bonds are often present in many active substances with different pharmacological effects, e.g., analgesic, anti-inflammatory, antimicrobial, or anticancer [8,12]. This may be due to the fact that they are neutral, stable, and have the properties of donating and accepting hydrogen bonds [13]. The aromatic compounds are relatively easy to synthesize from commercially available substrates, and the products are rather chemically stable. Our team has many years of experience in the synthesis and investigation of the multidirectional activity of various substituted benzamides, mainly anticancer, firstly netropsin analogs [14] and now trimethoprim derivatives [15].

**Figure 2:**
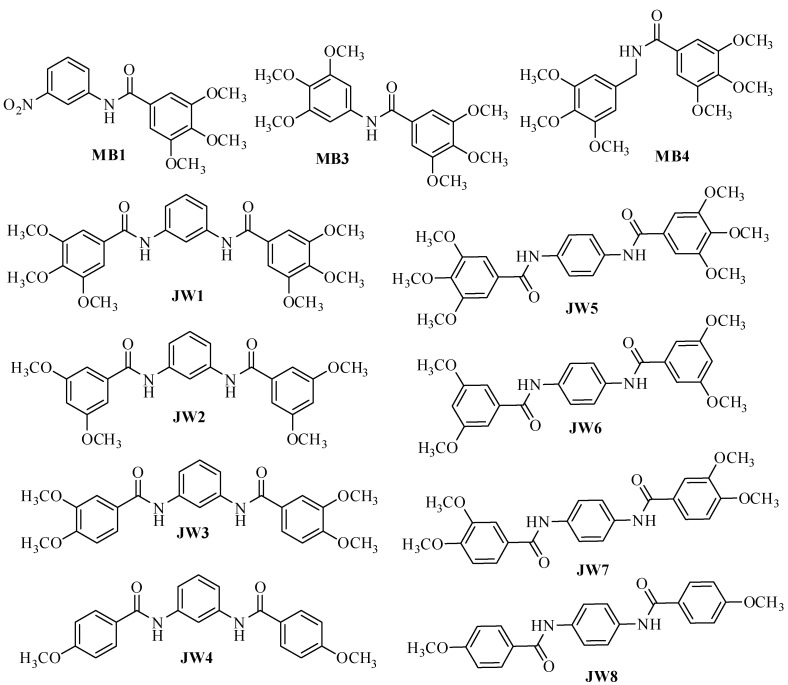
Structures of new benzamide DHFR inhibitors.

**Figure 3:**
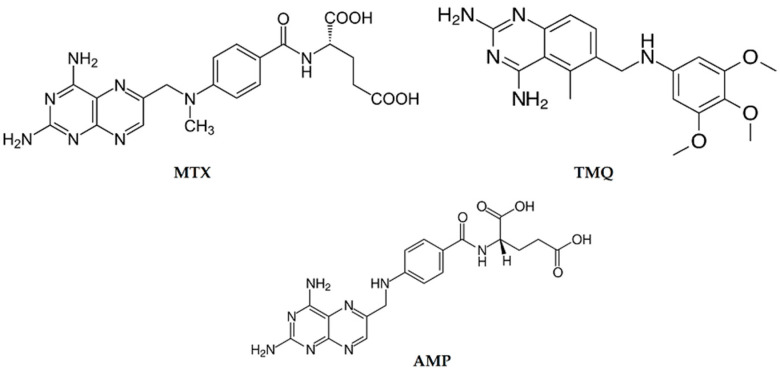
Structures of reference ligands.

The object of the research presented here is to investigate whether and how the number of benzene rings affects the inhibitory activity against *h*DHFR. We have recently synthesized a group of benzamide compounds (Figure 2) with interesting activity against enzymes involved in neurodegenerative processes [16]. Our group recently examined these compounds for their potential activity against Alzheimer’s disease [16]. Additionally, similar compounds were studied as potential agents against cancer [17] and Parkinson’s disease [18]. For the sake of comparison, the structures of the reference ligands (**MTX**—methotrexate, **AMP**—aminopterin, and **TMQ**—trimetrexate) are also given in Figure 3.

The structure of selected compounds suggests that they may inhibit reductase. It seems interesting to investigate whether and how elongation of the molecule by an additional amide bond and aromatic ring would influence the inhibitory effect against the enzyme. The absence of amino groups in the molecules raises further questions about the strength and mode of action of DHFR. Planned in vitro studies and molecular modeling provide us with the information we need.

## 2. Materials and Methods

### 2.1. General Information

All reagents were purchased from Fluka (Sigma-Aldrich, Poznań, Poland), Merck (Darmstadt, Germany), or Alfa Aesar (Karlsruhe, Germany) and used without further purification. Dichloromethane (DCM) and dimethylformamide (DMF) were stored in 4 Å molecular sieves. The Dihydrofolate Reductase Assay Kit was purchased from Sigma Aldrich (St. Louis, MO, USA).

### 2.2. Synthesis

The synthesis of the new compounds **JW1**–**JW8** and **MB1**, **MB3**, and **MB4** was carried out according to the protocol presented earlier [16].

### 2.3. Dihydrofolate Reductase (DHFR) Inhibition Assay

The inhibition of dihydrofolate reductase by the new **TMP** analogs, that is, determining their effect on the activity of recombinant human DHFR, was tested according to the instructions provided by the manufacturer and used previously [7]. Experiments were repeated three times; the calculated inhibitory concentrations of half the enzymatic activity, i.e., IC_50_ (µM), are included in Table 1.

### 2.4. Molecular Docking

Molecular docking was conducted using AutoDock Vina 1.2.5 software [19,20]. The *h*DHFR protein structure (PDB: 1U72, resolution: 1.90 Å [21]) and reference ligand structures **MTX** [22] and **TMQ** [23] were obtained from the PDB database. The search area was defined as a 20 × 20 × 20 Å cube. To accommodate variations in ligand–receptor interaction mechanisms, the analysis extended to the entire receptor area rather than focusing on specific fragments. This comprehensive approach proved particularly advantageous in scenarios like the absence of crystallographic data, which is actually a case for the proposed new inhibitors. Consequently, docking calculations were performed using a series of grid boxes covering the entire range of protein features. These boxes traversed the enzyme area, with the poses yielding the best scores automatically identified using an in-house script provided by the authors. This process iterated across subsequent grid boxes, ensuring the capture of the optimal ligand–receptor arrangement for all systems considered. The coordinates of the search area were shifted every two units within the range from x0 − 10 to x0 + 10, y0 − 10 to y0 + 10, and z0 − 10 to z0 + 10, with initial coordinates x0 = 30.00, y0 = 16.00, and z0 = 2.00. The in-house-developed scripts were used to move the docking box through the molecule. The EXHAUSTIVENESS parameter was set to 100 for more accurate calculations. The BIOVIA Discovery Studio Visualizer application was employed for visualizing the two-dimensional interactions between ligands and proteins [24]. The statistical analysis of molecular docking was carried out with the Statistica 13.0 program (StatSoft; Tulsa, OK, USA).

For the sake of validation of the docking procedure, a redocking process was carried out. The crystal structure of **MTX** taken from the PDB database was compared with the structure of this ligand after docking with the RMSD given by the following formula:

$$\mathrm{RMSD}=\sqrt{\frac{1}{N}\overset{N}{\underset{i=1}{\sum }}\delta_{i}^{2}}$$

*N*—number of atoms
*δ_i_—*distance between the *i*-th atom and the reference structure [Å].

**Figure 4:**
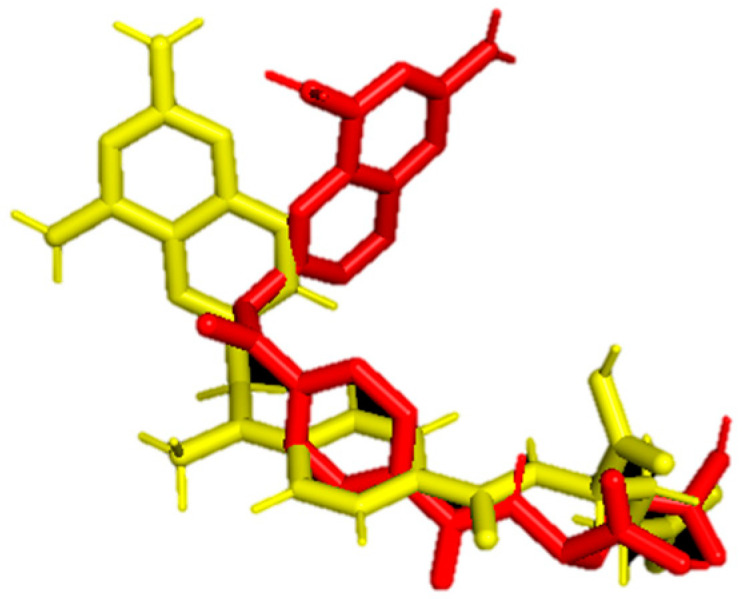
Comparison of the crystal structure of **MTX** (yellow) with the structure obtained after redocking (red).

To calculate RMSD for **MTX** crystal structure and post-docking structure, the Pymol program [25] was utilized. As shown in Figure 4, both conformations exhibit a satisfactory level of agreement with an RMSD of 2.142 Å.

### 2.5. Molecular Dynamics

The MD simulations were conducted using NAMD 2.14 [26] and VMD 1.9.3 [27] suites of programs, applying the CHARM22 force field. Input files were generated using the CHARM-GUI environment online [28,29]. During the preparation of the systems for simulation, they were solvated and ionized using 0.15 M NaCl. This enabled us to closely mimic the physiological conditions of the studied systems. Subsequently, prior to commencing the actual simulation, we conducted a preliminary one consisting of the following steps:

▪ Minimization: The MINIMIZE parameter was configured for 5000 steps, gradually heating the system from 0 K to 310 K.
▪ Equilibration lasting 1 ns.

Subsequently, a 20 ns simulation was carried out, with each step lasting 2 fs. Throughout this simulation, a constant temperature of 310 K and a constant pressure of 1 atm were maintained. The scripts provided by the VMD developers were employed to compute the parameters of the resultant trajectories. To uphold a pressure of 1 atm, the Langevin piston method was employed, featuring a decay period of 100 fs. Meanwhile, the constant temperature was upheld through Langevin dynamics, employing a damping factor of 5 (1/s).

### 2.6. ADMET Analysis

ADMETlab 2 was used to calculate ADMET parameters [30].

## 3. Results

### 3.1. In Vitro hDHFR Inhibitory Activity

The previously described method for evaluating the ability to inhibit *h*DHFR activity [7] allowed us to conclude that the tested benzamides are active in the range of IC_50_ values from 4.72 to 20.17 µM. All derivatives were more active than **TMP**, for which the IC_50_ value under the same conditions was 55.26 µM, but none of the benzamides showed higher activity than the reference **MTX** with an IC_50_ of 0.08 µM.

### 3.2. Statistical Analysis of hDHFR Test Results

In all experiments, the mean values for three assays ± standard deviations (S.D.) were calculated. The results were submitted to statistical analysis using the Student’s *t*-test. Differences were considered significant at *p* < 0.05.

### 3.3. Molecular Docking

Molecular docking was performed on **JW** and **MB** ligands, as well as reference ligands **MTX**, **TMQ**, and **AMP**, used in anticancer therapy [31,32,33]. The best-resulting affinities, together with IC_50_ values, are given in Table 1.

**Figure 5:**
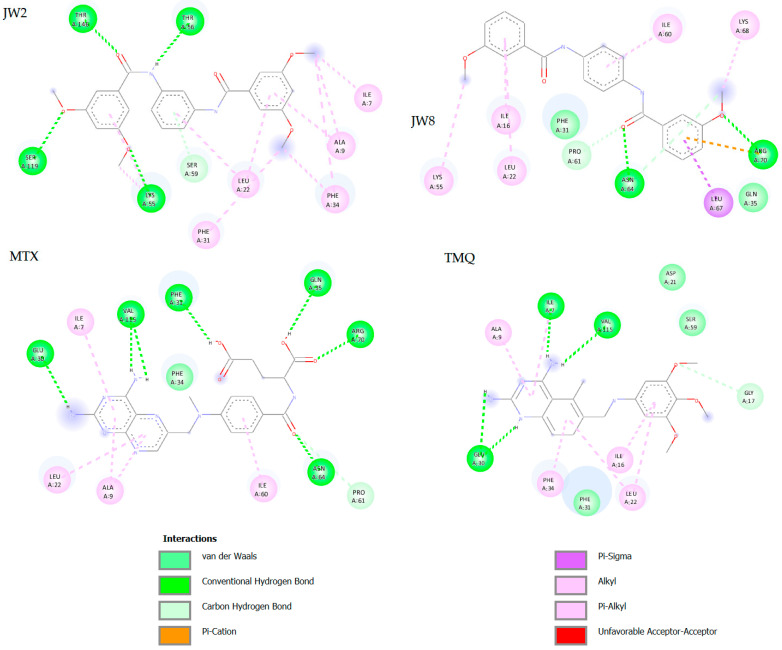
Two-dimensional diagrams of **JW2**, **JW8**, **MTX**, and **TMQ** interactions with *h*DHFR.

Interactions occurring between the ligand and the protein after docking were also analyzed are presented in Figure 5 [25].

### 3.4. Molecular Docking—Statistics

The preliminary docking revealed a significant variation in matches. In order to facilitate the data analysis, we have grouped matches for the two most promising ligands (**JW2** and **JW8**) and the reference compound (**TMQ**) into similar positions. For **JW2**, **JW8**, and **MTX**, 172 conformations each were analyzed and divided into five groups (Table 2). The number of items for each ligand are shown in Table 3 and comparison of median docking affinities for individual items in Table 4.

Due to significantly higher docking scores observed at positions 4 and 5 compared to those at positions 1–3, we focused our statistical analysis solely on the first three best locations. Position 1 consistently exhibited the lowest energy for each ligand, with **JW8** most frequently occupying this spot. For **JW2** and **MTX**, they were primarily found at positions 2 and 3, respectively. To assess discrepancies among individual poses, a Kruskal–Wallis test was conducted, revealing statistically significant differences (*p* < 0.05) in docking scores for **JW2**, **JW8**, and **MTX** at position 1. This trend was similarly observed for position 2 among these three ligands. Additionally, the energy at position 3 for **MTX** differed significantly from **JW2** and **JW8**, while there was no distinction between **JW2** and **JW8** (*p* > 0.05).

### 3.5. Molecular Dynamics

**Figure 6:**
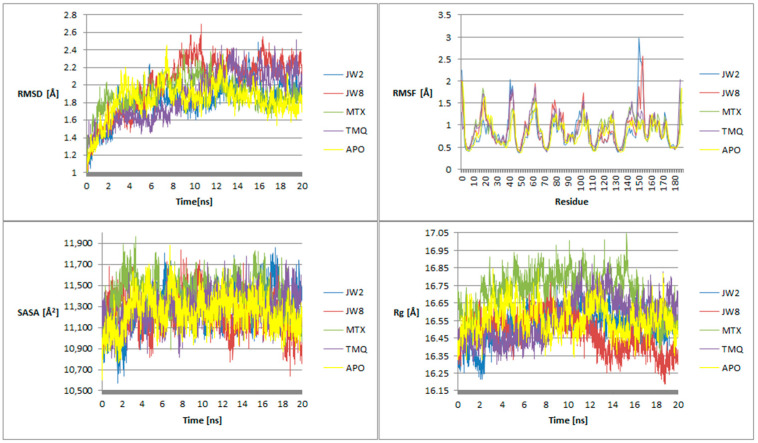
Parameter dependence diagrams: RMSD, RMSF, SASA, Rg for **JW2**, **JW8**, **MTX**, **TMQ** molecules, and apoenzyme on simulation time.

The molecular dynamics simulations, spanning 20 ns, scrutinized both the benzamide and reference ligands, along with the apoenzyme. The simulations commenced from the docking’s concluding position. Following the simulations, an examination was conducted on four parameters: RMSD, RMSF, SASA, and Rg (Figure 6).

**Figure 7:**
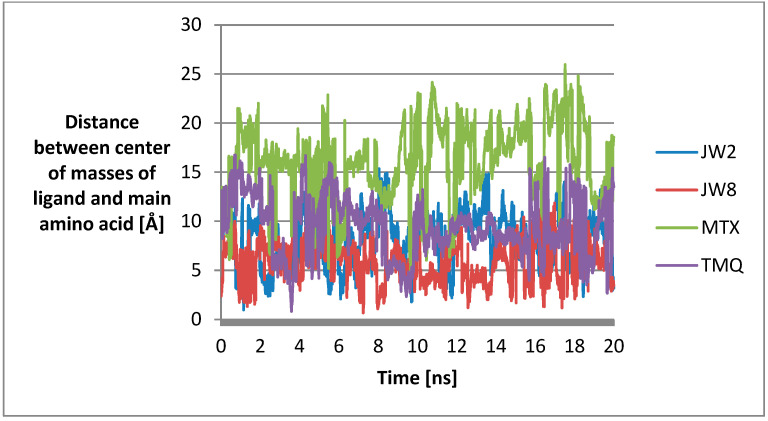
Plots of the dependence of the distance between the centers of masses of the ligand and the amino acid with which it forms hydrogen bonds have the highest percentage of frames on simulation time.

Hydrogen bonds between the ligand and the protein formed during the simulation were also analyzed. The results are summarized in Table 5 below:

To gain a deeper understanding of the mechanism of interaction of the most promising ligands with DHFR, we analyzed the time evolution of the distance of the centers of masses of the ligand and its key interacting aminoacid (i.e., the amino acid with which the ligand is in most frequent contact during the entire simulation, see Table 5), which provides a dynamic view of the binding process (Figure 7). This can help us understand how the ligand fluctuates in its binding pocket over time and how the interaction strength varies. Changes in the distance between the ligand and the amino acid can reflect alterations in the strength of hydrogen bonding interactions. Analysis of these fluctuations can provide insights into the binding energetics and the factors influencing ligand–receptor interactions.

Throughout the simulation, **JW8** consistently maintains the smallest distance from the amino acid Asp-64. The distances between **TMQ** and **JW2** and their respective residues are slightly larger and comparable. **MTX** exhibits the largest distance from the amino acid throughout the simulation, which suggests that this particular ligand may have weaker or less stable interactions with the amino acid compared to other ligands in the study. This indicates less favorable binding interactions between the ligand and the amino acid. Transient or unstable interactions with the amino acid lead to frequent fluctuations in distance throughout the simulation. This could suggest that the ligand’s binding interactions are less robust or less specific compared to other ligands.

### 3.6. ADMET Analysis

The ligands were analyzed for the values of a number of pharmacokinetic parameters in order to assess their potential use as drugs and to compare them with reference ligands (Table 6)

## 4. Discussion

### 4.1. In Vitro hDHFR Inhibitory Activity

The positive news from the received results is the achievement of activity greater than that of the model compound, trimethoprim. All of the tested benzamides are active against *h*DHFR, although they are less active than those described earlier and shown in Figure 2. However, we can, on the basis of the obtained results, supplement our knowledge of the structure–activity relationships, which will be able to be used in the design of further groups of active compounds. The first conclusion is that adding another benzene ring does not greatly increase the activity of the new derivatives. Similarly, the number of methoxy groups does not fundamentally change the inhibitory activity of the new derivatives. Moreover, looking at the activity of **MB** compounds, we see that the presence of the -NO_2_ group reduces the inhibitory activity of the derivatives—all compounds presented in Figure 2, with a similar structure to **MB1**, containing an -NH_2_ group and three methoxy groups, were more active. Also, the presence of additional methoxyl groups in **MB3** and **MB4** derivatives in place of the amine group did not result in greater inhibitory activity against *h*DHFR.

### 4.2. Molecular Docking

The binding energy between the ligands and the protein is notably lower for **JW2** and **JW8** compared to others. Although their IC_50_ values are not the smallest, they fall within an acceptable range. Consequently, **JW2** and **JW8** were chosen for further investigation alongside reference ligands **MTX** and **TMQ**. These selections were made due to the broader utility of these compounds in cancer therapy.

RMSD, or Root Mean Square Deviation, serves as a metric illustrating the average distance between atoms. The indicator typically exceeds only slightly beyond 2 Å, which is considered the upper limit of the acceptable RMSD parameter. In the case of **MTX**, RMSD values are slightly larger than 2 Å, which indicates the difference between conformation after docking and crystal structure obtained from PDB. All ligands interact with the protein through weak hydrophobic interactions. Except for **JW2**, they all form hydrogen bonds with carbon atoms. **JW8** interacts with amino acids in the protein through π–cation and π–σ interactions. Additionally, all ligands form hydrogen bonds with amino acids in the protein which descriptions are shown in Table 7.

**Figure 8:**
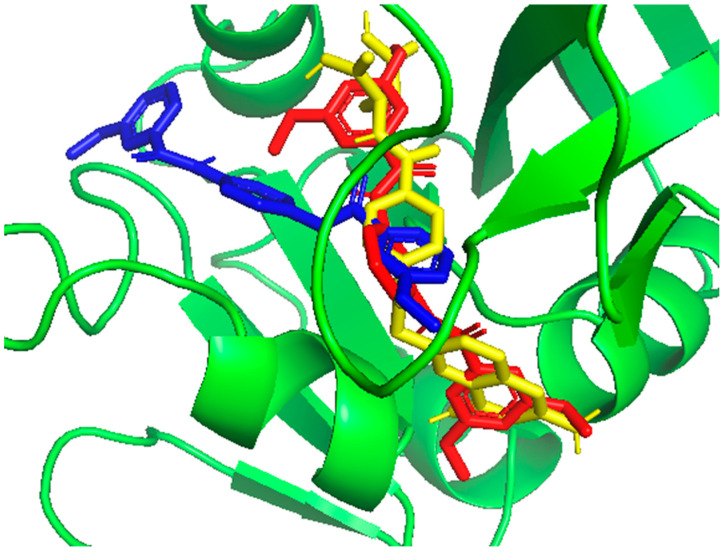
Structure of *h*DHFR with docked: **JW2** (red), **JW8** (blue), and **MTX** (yellow) with the lowest energies.

Reference ligands form more hydrogen bonds than **JW2** and **JW8** because of the lack of -NH_2_ groups in new benzamide molecules. This group is crucial to hydrogen bond formation in the case of **MTX** and **TMQ**, but **JW2** and **JW8** are forming hydrogen bonds mainly due to the presence of amide or methoxyl groups. Analysis of the molecular docking results shows that **JW2** and **JW8**, as well as **MTX**, bind to hDHFR in different ways. This is due to different interactions with the protein, among other things, the different number of hydrogen bonds, and the fact that these bonds are not formed with the same amino acids. These conclusions are supported by statistical docking analysis, which shows statistically significant differences between the binding energies for **JW2**, **JW8**, and **MTX** (see Figure 8 below).

### 4.3. Molecular Dynamics

The RMSD trends across the simulation duration reveal distinct behaviors among the ligands. For **JW2**, initially, its RMSD values closely mirror those of the apoenzyme, gradually decreasing thereafter before rising above the apoenzyme’s values around 14 ns. In comparison to **MTX**, **JW2** exhibits lower RMSD values until approximately 12 ns, after which they become comparable. At the onset of the simulation, TMQ displays smaller RMSD values than **JW2**; however, around 9 ns, their values begin to converge, with **JW2** occasionally exhibiting smaller values thereafter. Regarding **JW8**, its RMSD values at the simulation’s onset are akin to those of the apoenzyme and **TMQ**, yet smaller than **MTX**. Between 2 and 4.5 ns, **JW8**’s values are comparable to those of **MTX** and **TMQ** but lower than the apoenzyme’s. From 4.5 to 12 ns, **JW8**’s RMSD peaks exceed those of other ligands, and beyond 12 ns, they become comparable to **TMQ** but remain higher than the apoenzyme’s. These findings suggest that **JW2** tends to stabilize the system to a greater extent than **JW8**.

**JW2** is characterized by a low RMSF value compared to the apoenzyme, **MTX**, and **TMQ**, with exceptions observed for a few amino acids such as serine 42, glutamic acid 44, and isoleucine 151. In contrast, **JW8** exhibits comparable or lower RMSF values compared to the apoenzyme, **MTX**, and **TMQ**. Notably, exceptions are observed for amino acids, including lysine 63, glutamic acid 104, isoleucine 151, and glutamic acid 154. **JW2** forms a more stable arrangement than the apoenzyme, while **JW8**’s stability is comparable to that of the apoenzyme.

Throughout the simulation, **JW2** consistently exhibits a low SASA parameter value compared to both the reference ligands and the apoenzyme, except during the intervals of approximately 5.5–6.5 ns and 16.5–17.5 ns. On the other hand, **JW8** generally maintains a comparable SASA parameter value to the reference ligands and the apoenzyme, except during the intervals of around 3.5–4.5 ns, 11.5–13.5 ns, and from approximately 18 ns until the end, where it records the lowest values among all analyzed ligands. However, during intervals approximately spanning 0.5–1 ns, 7.5–8.5 ns, and about 9.75 ns, **JW8** registers values higher than those of the apoenzyme, **MTX**, and **TMQ**. These findings underscore the greater stability of both **JW2** and **JW8** systems compared to the apoenzyme and reference ligands.

Throughout the simulation, the Rg parameter for **JW2** remains consistently lower or comparable to that of the apoenzyme and **TMQ** and lower than that of **MTX**. Particularly in the initial phase of the simulation, spanning up to about 3 ns, **JW2** exhibits the lowest Rg value among all tested ligands and the unliganded enzyme. In contrast, **JW8** maintains a lower Rg value than **MTX** for approximately half of the simulation duration, slightly higher than **TMQ**, and comparable to the apoenzyme. However, in the latter portion of the simulation, Rg values for **JW8** become the lowest among all analyzed ligands and the apoform. Analysis of the Rg parameter values indicates the stability of both **JW2** and **JW8** systems.

The stability of the **JW2** and **JW8** circuits becomes evident during simulation, with **JW2** demonstrating greater stability compared to **JW8**. This contrast is particularly noticeable in the latter half of the simulation, where systems containing reference ligands exhibit more pronounced chaotic behavior.

In addition, the stability of systems with benzamide ligands is revealed in the distances between the amino acid with which the most common hydrogen bonds are formed and the ligand (Figure 7). For **JW8**, it is the smallest, and for **JW2**, it is on par with **TMQ** and smaller than **MTX**. In addition, the changes in measured distances are more chaotic for **MTX** and **TMQ** compared to **JW8** and **JW2**.

The key hydrogen bonds established by the reference ligands primarily involve the Glu-30 residue [7]. These bonds arise due to the amino group present in the structure of these ligands. Conversely, **JW2** and **JW8** lack this group, leading to the absence of hydrogen bonding with Glu-30. This indicates a distinct mechanism of *h*DHFR enzyme inhibition compared to **MTX** and **TMQ**. Benzamide ligands form fewer hydrogen bonds with shorter durations compared to reference ligands. Notably, **JW2** exhibits more pronounced differences between bonds detected during docking and simulation stages compared to **JW8**. The most notable binding in **JW2** involves the Gly-117 residue, whereas in **JW8**, it involves Asn-64 and Arg-70.

### 4.4. ADMET Analysis

ADMET analysis was conducted on the benzamide derivatives **JW2** and **JW8**, alongside **MTX** and **TMQ** as reference ligands. The benzamide derivatives exhibited low to medium toxicity in rats, akin to **MTX** but less than **TMQ**. All four compounds demonstrated low carcinogenicity. Notably, **JW2** and **JW8** displayed lower hepatotoxicity compared to the reference ligands, suggesting promise, particularly considering the liver-related symptoms associated with methotrexate [34]. The benzamide ligands exhibit medium to high skin toxicity, with **MTX** showing comparatively lower toxicity in this regard. **TMQ** demonstrates high inhalation toxicity, while the other ligands display low toxicity. Additionally, **TMQ** exhibits high AMES activity, whereas the benzamide ligands demonstrate low to medium AMES activity. All substances analyzed for ADMET show low ocular toxicity. Both **JW8** and **TMQ** adhere to all rules, including Lipinski, Pfizer, GSK, and Golden Triangle, whereas **JW2** only fails to meet the GSK rule. Conversely, **MTX** complies with only the Pfizer and Golden Triangle rules. Both benzamide ligands and reference ligands exhibit low intestinal absorption and a low F20%. **JW2** and **JW8** show higher plasma protein binding compared to **MTX**. The benzamide ligands and **MTX** display low blood–brain barrier permeability, while **TMQ** shows medium permeability. **JW2** and **JW8** have a high likelihood of prostaglandin H2 inhibition but a low likelihood of being substrates. The opposite trend is observed for the reference ligands. According to the ADMET analysis, **JW2** and **JW8** hold potential for drug use, with several parameters closely resembling those of the reference ligands and many being more favorable, such as hepatotoxicity, inhalation toxicity, AMES activity, and rat toxicity. Less favorable parameters include plasma protein binding and prostaglandin H2 inhibition. The ADMET analysis suggests that both **JW2** and **JW8** could potentially serve as drugs [35].

## 5. Conclusions

In recent years, there has been growing interest in antifolates in cancer chemotherapy, which has prompted the medicinal chemistry community to develop new and selective inhibitors of human DHFR. Both **JW2** and **JW8** hold promise for application in cancer therapy. This potential is supported by their favorable IC_50_ values and lower ligand–protein binding energy compared to reference ligands. It is worth noting that benzamide ligands operate through a distinct mechanism from reference ligands, as they do not engage in hydrogen bonding with the Glu-30 residue, unlike **MTX** and **TMQ**. In **JW2**, the key residue is Gly-117, while for **JW8**, it is Asn-64 and Arg-70. Benzamide ligands exhibit reduced hepatotoxicity compared to **MTX** and **TMQ**, albeit with a higher plasma protein binding rate. Furthermore, **JW2** and **JW8** demonstrate the ability to stabilize the *h*DHFR enzyme, despite forming fewer hydrogen bonds with the protein compared to reference ligands, as observed in the parameters analyzed during MD simulations. Reduced stability of MTX–DHFR interactions may contribute to the development of drug resistance if cancer cells adapt to evade the effects of the ligand. In such cases, strategies to enhance ligand stability or modify the ligand structure to improve binding affinity are necessary to overcome resistance and restore anticancer activity. As observed in the parameters analyzed during MD simulations, **JW2** and **JW8** demonstrate the ability to stabilize the *h*DHFR enzyme despite forming fewer hydrogen bonds with the protein compared to reference ligands. Since cells can adapt to changes in ligand–receptor interactions by altering expression levels of downstream effectors or activating compensatory signaling pathways, this feature is very encouraging in the context of the anticancer properties of these compounds.

This paper introduces a novel group of *h*DHFR inhibitors, which expands research in the field of potential anticancer drugs. These structures may have an interesting future as a template for developing new analogs with potential anticancer properties. Confirmation of the desirability of testing on cancer cells is provided by the results of a study in which compounds **MB3** and **MB4**, synthesized by a different method, were initially tested against various cancer cells and showed antiproliferative activity [18]. In order to confirm the activity of the described compounds, we plan to do further in vitro investigations of their activity on cancer cell lines to confirm their effectiveness and potential use in therapeutic applications.

## Figures and Tables

**Table 1:** Comparison of the most favorable values of ligand–protein binding energy with the IC_50_ ratio.

Ligand	Energy [kcal/mol]	IC_50_ [μM]
**JW1**	−9.377	8.50 ± 0.06
**JW2**	−10.11	10.15 ± 0.02
**JW3**	−9.379	9.83 ± 0.03
**JW4**	−9.988	15.30 ± 0.04
**JW5**	−9.069	4.80 ± 0.05
**JW6**	−9.973	4.72 ± 0.05
**JW7**	−9.719	8.13 ± 0.06
**JW8**	−10.01	12.15 ± 0.02
**MB1**	−7.974	10.16 ± 0.01
**MB3**	−7.267	9.17 ± 0.01
**MB4**	−7.713	20.17 ± 0.03
**MTX**	−9.525	0.08 ± 0.03
**TMQ**	−9.655	n.d.
**AMP**	−9.871	n.d.
**TMP**	-	55.26 ± 0.01

**Table 2:** The most important docking poses of **JW2**, **JW8**, and **MTX**.

	JW2	JW8	MTX
Position 1	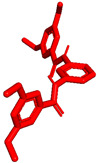	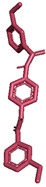	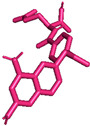
Position 2	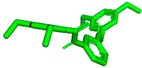	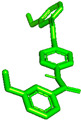	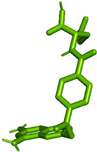
Position 3	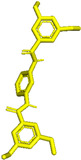	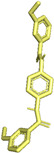	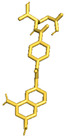
Position 4	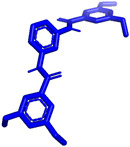	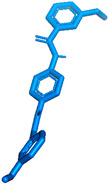	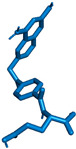
Position 5	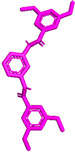	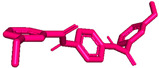	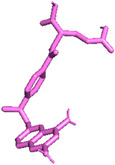

**Table 3:** The number of items for each ligand.

	JW2	JW8	MTX
Position 1	51 (29.65%)	89 (51.74%)	22 (12.79%)
Position 2	12 (6.97%)	24 (13.95%)	99 (57.56%)
Position 3	61 (35.47%)	27 (15.7%)	7 (4.07%)
Position 4	40 (23.26%)	26 (15.12%)	30 (17.44%)
Position 5	8 (4.65%)	6 (3.49%)	14 (8.14%)

**Table 4:** Comparison of median docking affinities for individual items [kcal/mol]. The values of the minimum and maximum energy of a given item (min/max) are also given, and the normality of the distribution of the energy values of each item was checked using the W Shapiro–Wilk test: *p* < 0.05—the distribution is not normal; *p* > 0.05—the distribution is normal.

	JW2	JW8	MTX
Position 1	−9.747 (−10.11/−9.009) *p* < 0.05	−9.9 (−10.01/−9.136) *p* < 0.05	−9.4145 (−9.525/−8.539) *p* < 0.05
Position 2	−9.1505 (−9.333/−6.593) *p* > 0.05	−9.4855 (−9.556/−9.259) *p* < 0.05	−9.345 (−9.453/−7.928) *p* < 0.05
Position 3	−9.039 (−9.2/−8.109) *p* < 0.05	−9.252 (−9.329/−6.403) *p* < 0.05	−9.332 (−9.351/−9.235) *p* > 0.05
Position 4	−8.02 (−8.871/−6.831) *p* > 0.05	−8.9275 (−9.199/−6.292) *p* < 0.05	−8.9475 (−9.948/−8.82) *p* < 0.05
Position 5	−7.9775 (−8.61/−4.979) *p* > 0.05	−7.6465 (−9.001/−6.088) *p* < 0.05	−8.6855 (−8.899/−7.843) *p* < 0.05

**Table 5:** A summary of the hydrogen bonds involving ligands **JW2**, **JW8**, **MTX**, **TMQ**, and *h*DHFR is provided, considering the donor, acceptor, percentage of instances where the hydrogen bond is present, and the type of chain (main and side) contributing to its formation.

Ligand	Hydrogen Bond Donor	Hydrogen Bond Acceptor	Percentage of Instances with Hydrogen Bonding [%]
**JW2**	GLY117—main	JW2—side	13.85
	JW2—side	THR56—side	3.70
**JW8**	JW8—side	ASN64—main	14.70
	JW8—side	SER59—side	5.75
	GLN35—side	JW8—side	5.45
	ARG70—side	JW8—side	2.55
**MTX**	MTX—side	GLU30—side	36.40
	MTX—side	ALA9—main	34.25
	ALA9—main	MTX—main	33.55
**TMQ**	TMQ—side	GLU30—side	32.70
	TMQ—side	ILE7—main	16.95
	TMQ—side	ASP21—side	7.40
	TMQ—side	VAL115—main	3.30
	THR56—side	TMQ—side	1.90

**Table 6:** Pharmacokinetic parameters for benzamide and reference ligands (H—high, M—medium, L—low, Y—yes, N—no). (HIA—human intestinal absorption; F_20%_—human oral bioavailability 20%; PPB—plasma protein binding; BBB—blood–brain barrier penetration; PGHinh/Pghsub—probability of being PGH inhibitor/substarte).

Ligand	Oral Toxicity for Rats	Carcinogenicity/ Hepatotoxicity/ Dermal Toxicity/ Inhalation Toxicity/AMES/ Eye Toxicity	Principle: Lipinski/Pfizer/GSK/Golden Triangle	HIA/F_20%_/PPB/ BBB/PGHinh/ PGHsub
**JW2**	L	L/L/M/L/L/L	Y/Y/N/Y	L/L/H/L/H/L
**JW8**	M	L/L/H/L/M/L	Y/Y/Y/Y	L/L/H/L/H/L
**MTX**	L	L/H/L/L/L/L	N/Y/N/Y	L/L/L/L/L/H
**TMQ**	H	L/H/H/H/H/L	Y/Y/Y/Y	L/L/H/M/H/H

**Table 7:** Description of conventional hydrogen bonds between ligands **JW2**, **JW8**, **MTX**, **TMQ**, and *h*DHFR.

Ligand	Amino Acid		Location of Hydrogen Bond in the Inhibitor Molecule
	Name	Number	
**JW2**	Lysine	55	the hydrogen atom in the amide group
	Threonine	56	the oxygen atom in the methoxy group
	Serine	119	the oxygen atom in the methoxy group.
	Threonine	146	the oxygen atom in the amide group
**JW8**	Asparagine	64	the oxygen atom in the amide group
	Arginine	70	the oxygen atom in the methoxy group an additional π–cation interaction with the aromatic ring
**MTX**	Glutamic acid	30	the -NH_2_ group
	Phenylalanine	31	the -OH group in the carboxyl group
	Glutamine	35	the -OH group in the carboxyl group
	Asparagine	64	the C=O group in the amide group
	Arginine	70	the C=O group in the carboxyl group
	Valine	115	the -NH_2_ group
**TMQ**	Isoleucine	7	the -NH_2_ group
	Glutamic acid	30	the -NH_2_ group
	Valine	115	the -NH_2_ group
	Tyrosine	121	the -NH_2_ group
	Threonine	136	the -NH_2_ group
	Threonine	146	the methoxyl group

## Data Availability

Data are contained within the article.
